# Case of MYB-Rearranged Prostatic Adenoid Cystic Carcinoma

**DOI:** 10.3390/curroncol33090527

**Published:** 2026-09-01

**Authors:** Sha Liu, Yuhan Liu, Ziyu Zhang, Shuiping Yin, Xinyi Wu, Ying Dai, Yingying Du

**Affiliations:** 1 Department of Oncology, The First Affiliated Hospital of Anhui Medical University, Hefei 230022, China; 2 First Clinical Medical College, Anhui Medical University, Hefei 230032, China; 3 Department of Urology, The First Affiliated Hospital of Anhui Medical University, Hefei 230022, China; 4 Anhui Province Key Laboratory of Genitourinary Diseases, Anhui Medical University, Hefei 230022, China; 5 Institute of Urology, Anhui Medical University, Hefei 230022, China; 6 Department of Oncology, The Fifth Affiliated Hospital of Anhui Medical University, Fuyang 236000, China

**Keywords:** prostatic adenoid cystic carcinoma, basal cell carcinoma, MYB gene rearrangement, molecular classification, platinum-based chemotherapy

## Abstract

**Background:** Prostatic adenoid cystic carcinoma/basal cell carcinoma (ACC/BCC) has been reclassified under the fifth edition of the World Health Organization’s classification of tumors, distinguishing it from basal cell cancer of the skin. This malignant neoplasm exhibits distinct biological characteristics that differ from those of typical prostatic adenocarcinoma. However, optimal clinical management of prostatic ACC/BCC remains uncertain because of its rarity and the limited evidence available. **Methods:** This study retrospectively reviews the treatment course of a 62-year-old male patient presenting with more than six months of dysuria. Initial management included transurethral plasmakinetic resection of the prostate (TUPKP), followed by robot-assisted radical prostatectomy and bilateral pelvic lymph node dissection. Postoperative fluorescence in situ hybridization (FISH) demonstrated MYB rearrangement, providing molecular support for the pathological classification of prostatic ACC/BCC and facilitating diagnostic reclassification. **Results:** Pathological examination of the TUPKP specimen indicated poorly differentiated carcinoma, with findings consistent with prostatic ACC/BCC. Preoperative imaging showed an irregular soft-tissue lesion in the prostate/bladder neck region, without definite pelvic lymph node or distant organ metastasis. Histological analysis demonstrated cribriform structures and perineural invasion, while immunohistochemistry supported a basal cell phenotype; together with these findings, detection of MYB rearrangement via FISH supported reclassification of the tumor as prostatic ACC/BCC. Following radical surgery, adjuvant paclitaxel plus carboplatin was administered as an individualized empirical treatment in the absence of an established disease-specific standard. The patient completed six cycles of adjuvant chemotherapy and remained clinically stable during follow-up, with no radiological evidence of recurrence at the latest evaluation. **Conclusions:** This case highlights the diagnostic challenges of prostatic ACC/BCC and underscores the value of integrating molecular findings with histopathological and immunohistochemical features to support accurate tumor classification and individualized clinical management. MYB rearrangement may provide useful molecular support for diagnosis and classification; however, its biological and potential therapeutic significance in prostatic ACC/BCC requires further investigation in larger cohorts.

## Simple Summary

Prostatic adenoid cystic/basal cell carcinoma is an exceptionally rare malignancy that can mimic more common prostate tumors, creating important diagnostic and therapeutic challenges. We report a 62-year old man initially managed as having high-risk conventional prostate cancer. Reassessment of the pathological and immunohistochemical findings, together with detection of an MYB rearrangement via fluorescence in situ hybridization, supported reclassification of the tumor as prostatic adenoid cystic/basal cell carcinoma and prompted reconsideration of the initial diagnostic and management approach. Following radical surgery, adjuvant paclitaxel plus carboplatin was administered as an individualized empirical treatment in the absence of an established disease-specific standard. No definite recurrence or metastasis was detected during approximately nine and a half months of postoperative follow-up. This case highlights the value of integrated pathological and molecular assessment in diagnostically challenging prostate tumors while underscoring the need for further evidence to define optimal management strategies and long-term outcomes.

## 1. Introduction

In 2022, prostatic adenoid cystic/basal cell carcinoma was officially recognized by the World Health Organization (WHO) Classification of Tumors of the Urinary and Male Genital Systems as a malignant tumor of the prostate derived from prostatic basal cells [1,2]. This condition exhibits several morphological patterns, including cribriform, trabecular, or solid small nests, and is no longer included in the Gleason grading system [3].

These subtypes constitute an extremely small proportion of the overall prostate cancer population, reported to be less than 0.1%. However, these tumors can exhibit local recurrence and distant metastasis to the lungs and bones [3]; therefore, their biological behavior should not be considered indolent. Most patients present with lower urinary tract symptoms or hematuria, the imaging findings are generally nonspecific, and their prostate-specific antigen (PSA) in serum can increase slightly or be within the normal range; therefore, this type of tumor is prone to being diagnosed as benign prostatic hyperplasia or conventional adenocarcinoma.

At the molecular level, MYB-NFIB (nuclear factor I/B) fusion proteins and other structural abnormalities of MYB have been identified as crucial drivers for adenoid cystic carcinoma (ACC) transformation in salivary glands [4]. MYB rearrangements, including MYB–NFIB fusion, have also been identified in a subset of prostatic basal cell carcinomas, particularly those with adenoid cystic carcinoma-like morphology, and provide additional molecular support for classification of these tumors [5]. However, optimal treatment strategies for prostatic ACC/BCC remain poorly defined because of the rarity of the disease and the limited clinical evidence available.

Based on integrated imaging, pathological, and molecular findings, this case report aims to describe the diagnostic process leading to tumor reclassification and the subsequent individualized management of this rare entity.

## 2. Case Presentation

### 2.1. Basic Information

The patient was a 62-year-old male. He had a two-year history of hypertension, with the highest documented blood pressure of 140/100 mmHg; his blood pressure had returned to normal without antihypertensive medication. He had previously undergone spinal internal fixation with retained hardware. He had no family history of malignancy, chronic toxin or radiation exposure, and no other relevant medical history.

### 2.2. Course at Outside Institutions

Around six to seven months prior to admission, the patient complained of lower abdominal pain, decreased urinary flow, and difficulties in performing daily activities. A few days later, he had persistent mild pain in the lower right abdomen and lower back.

Evaluation at the local hospital showed a degree of prostatic enlargement, which was diagnosed as benign prostatic hyperplasia with potential tumor. On 4 July 2025, the patient underwent transurethral plasmakinetic resection of the prostate (TUPKP).

The routine pathology of the TUPKP specimen was poorly differentiated carcinoma. Immunohistochemistry showed that P63 and CK5/6 were expressed diffusely, and atypical PSA expression was also detected. Consultation led to the proposal of prostatic ACC/BCC and radical operation was recommended after molecular testing.

Meanwhile, considering the presence of high-risk prostatic adenocarcinoma, androgen deprivation therapy was administered at this time, including 3.6 mg of goserelin every four weeks combined with 160 mg of enzalutamide per day.

### 2.3. Admission and Imaging Evaluation

On 18 August 2025, the patient was admitted to the Department of Urology of our hospital with a history of TUPKP for prostatic malignancy.

Physical examination revealed no significant abnormalities. ECOG performance status score was grade 1. Baseline laboratory evaluation, including complete blood count, coagulation profile, liver function, renal function, and serum PSA, showed no clinically significant abnormalities. Representative baseline laboratory findings are summarized in Table 1.

**Figure 1:**
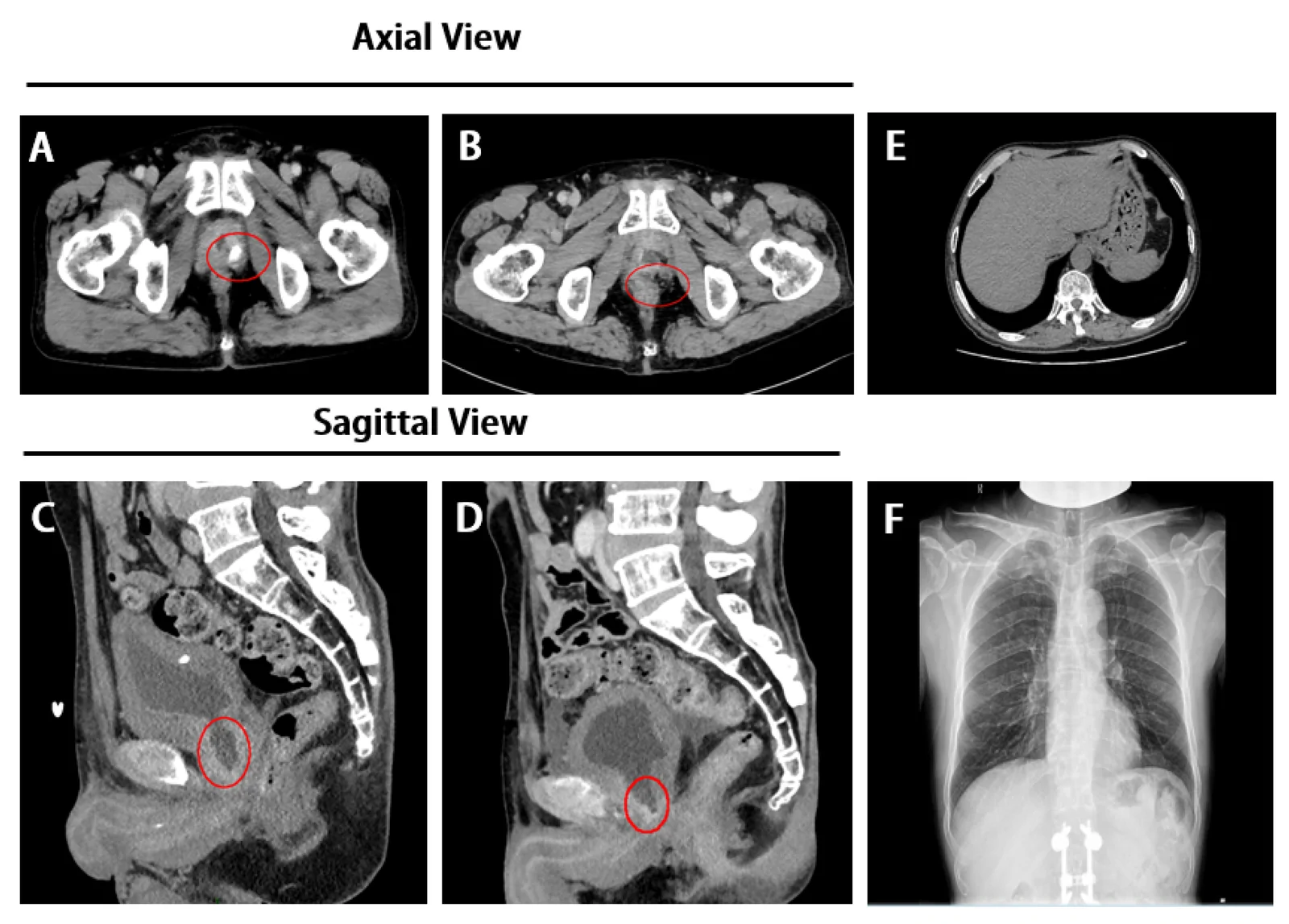
Preoperative contrast-enhanced pelvic computed tomography (CT) images, including axial (**A**) and sagittal (**C**) views, revealed an irregular soft-tissue lesion in the prostate/bladder neck region (red circles). Postoperative follow-up pelvic CT images, including axial (**B**) and sagittal (**D**) views, showed no definite residual nodular lesion in the corresponding region (red circles). Preoperative upper abdominal CT (**E**) revealed no focal hepatic lesion or other evidence of upper abdominal metastasis. Preoperative chest radiography (**F**) showed no focal pulmonary lesion suspicious for metastasis.

Bone scintigraphy showed no osseous metastasis. Preoperative contrast-enhanced pelvic CT demonstrated irregular soft-tissue thickening in the prostate/bladder neck region, with mild heterogeneous enhancement adjacent to the bladder neck. No definite pelvic lymph node metastasis or distant organ metastasis was identified (Figure 1A).

Postoperative follow-up pelvic CT showed no definite residual nodular lesion in the previously involved region (Figure 1B).

Preoperative chest radiography showed no focal pulmonary lesion suspicious for metastasis (Figure 1C), while upper abdominal CT revealed no focal hepatic lesion or other evidence of upper abdominal metastasis (Figure 1D). Before admission to our institution, the patient also underwent 18F-fluorodeoxyglucose positron emission tomography/computed tomography (18F-FDG PET/CT) at Fudan University Shanghai Cancer Center. According to the official report, increased FDG uptake was observed in the prostate, with bilateral iliac and presacral lymph nodes considered suspicious for metastasis; however, no distant metastatic lesion was reported. As the examination was performed at an outside institution, the original PET/CT images were unavailable for retrospective review, and only the official written report could be retrieved. Therefore, these lymph node findings represent radiological suspicion rather than pathological confirmation.

### 2.4. Surgical Procedure

Following a multidisciplinary team (MDT) meeting, radical surgery was deemed necessary due to the high risk of recurrence and the absence of distant metastasis.

On 25 August 2025, a robotic-assisted laparoscopic radical prostatectomy with bilateral pelvic lymphadenectomy was performed using a transabdominal approach under pneumoperitoneum. The operation was started at 12:50 and finished at 16:25, with a total operative time of about 215 min and an estimated blood loss of 50 mL. During the operation, the plane between the bladder neck and the prostate was not clear and the suspicious bladder neck tissue was sent separately for pathology. The prostate and seminal vesicles were removed en bloc. There was no active bleeding or rectal injury at the end of the procedure. Bilateral pelvic lymph node dissection was carried out according to the standard pelvic lymph node dissection template for prostate cancer, along the external iliac vessels and within the obturator fossa, including lymphatic tissue surrounding the obturator nerves and extending inferiorly to the Cloquet node region. The presacral lymph node region was not included in the dissection field, consistent with the routine surgical approach.

### 2.5. Postoperative Pathology and Immunohistochemical Staining

The initial TUPKP specimen and the subsequent radical prostatectomy specimen were jointly re-reviewed by two experienced genitourinary pathologists to confirm the histopathological diagnosis. Approximately 6 × 4 × 3 cm of the radical prostatectomy specimen was obtained. A gray-white solid nodule was seen in both lobes of the prostate, mainly affecting the left lobe, with foci of capsule penetration and spread to the bladder neck.

**Figure 2:**
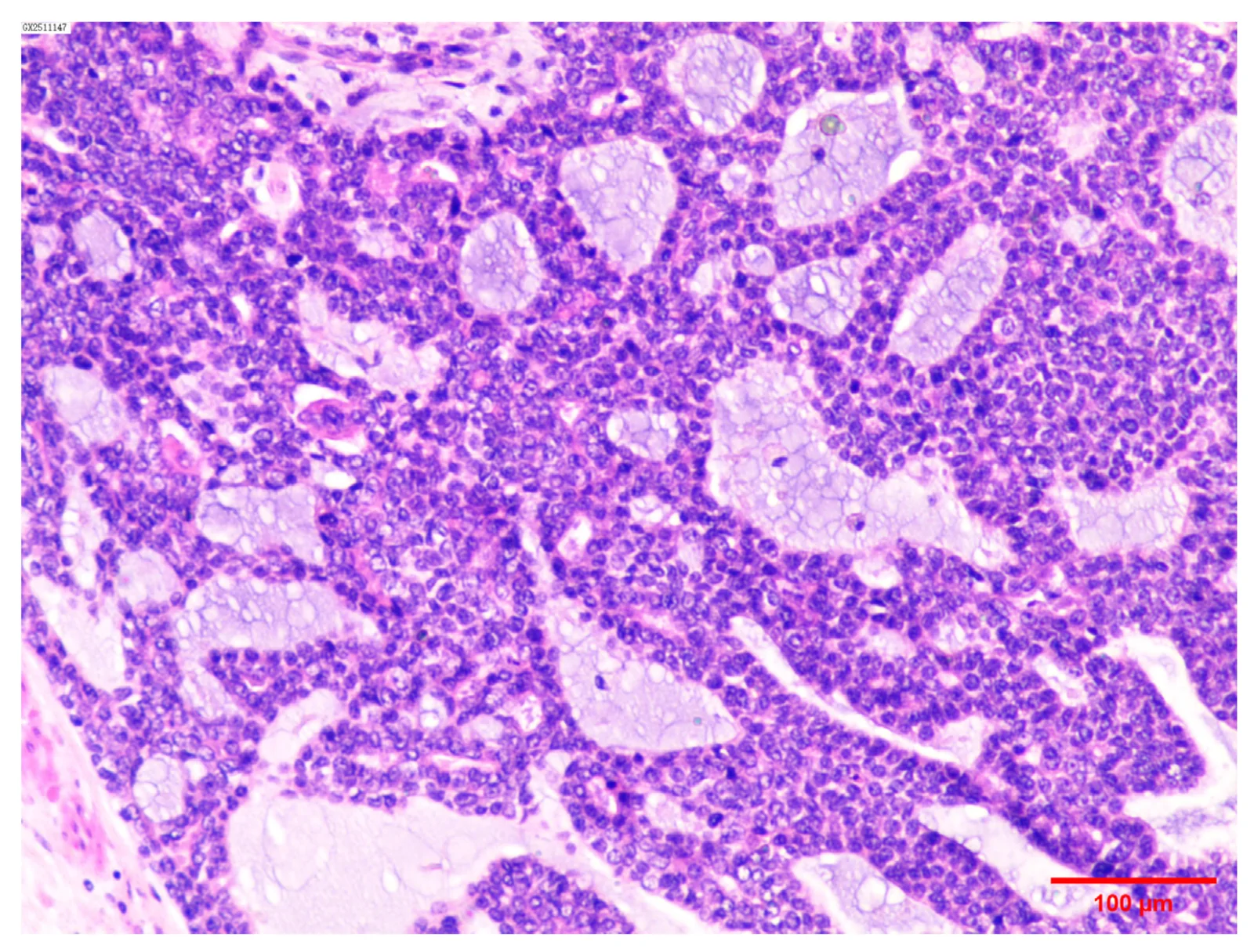
Histopathological architecture of the tumor. Hematoxylin and eosin staining showed small basaloid tumor cells with hyperchromatic nuclei and a high nuclear-to-cytoplasmic ratio, arranged predominantly in cribriform, glandular, and solid nest-like structures within a fibrous stroma. Some lumina contained eosinophilic or mucinous secretions. Original magnification ×200; scale bar = 100 μm.

**Figure 3:**
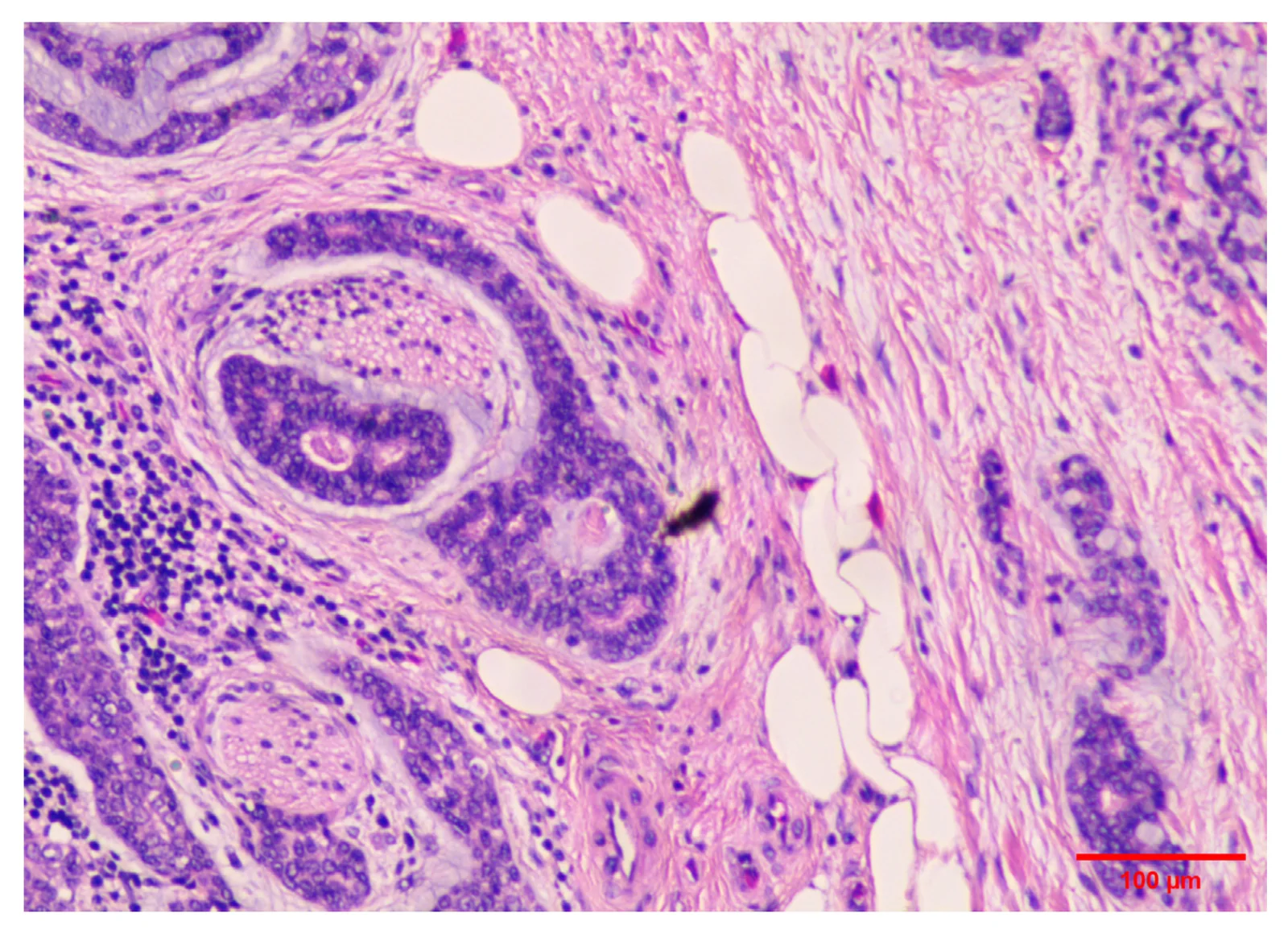
Hematoxylin and eosin staining demonstrating perineural invasion. Tumor cells are observed surrounding and infiltrating nerve structures within the tumor stroma. Original magnification ×200; scale bar = 100 μm.

Microscopic examination (Figure 2 and Figure 3) showed small tumor cells with hyperchromatic nuclei and a high nuclear-to-cytoplasmic ratio, arranged in cribriform, glandular, and solid nest structures within fibrous stroma. Some lumina contained eosinophilic or mucinous material. Perineural invasion and submucosal invasion of the bladder neck were present without seminal vesicle involvement.

A total of 15 dissected pelvic lymph nodes were examined, including 6 left-sided and 9 right-sided lymph nodes, and none showed metastatic involvement (0/15).

**Figure 4:**
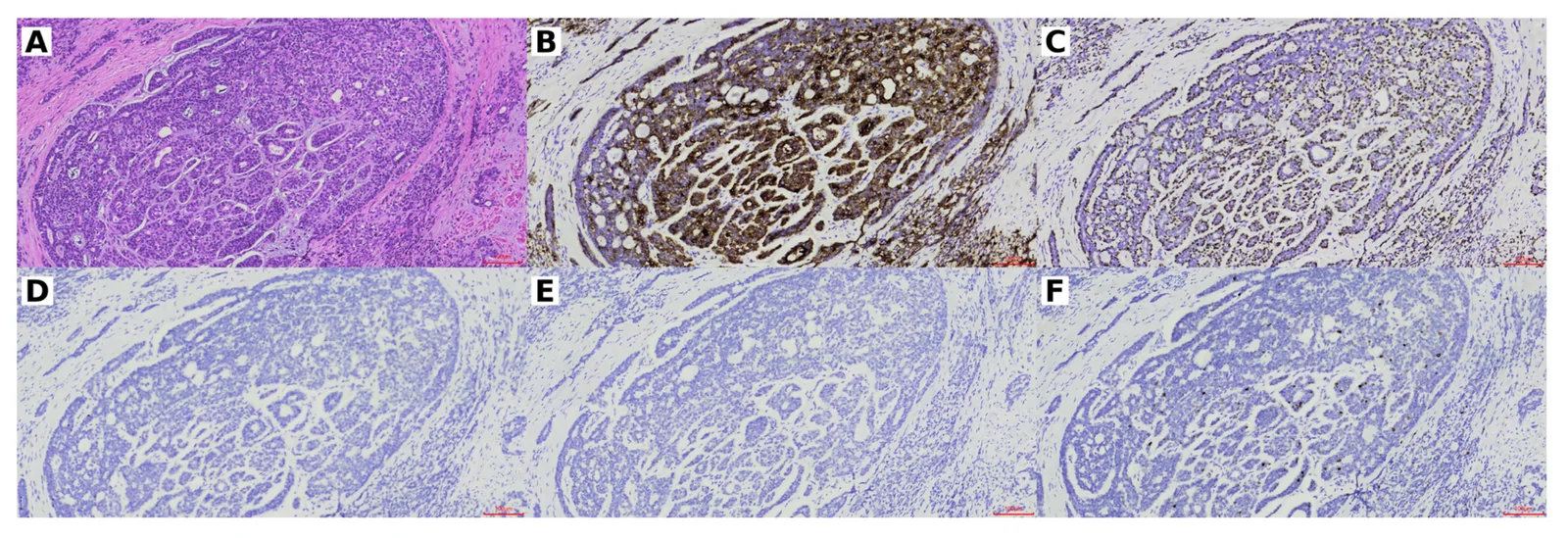
Histopathological and immunohistochemical features of prostatic adenoid cystic/basal cell carcinoma. (**A**) Hematoxylin and eosin staining demonstrated a mixed adenoid cystic/cribriform and basaloid growth pattern. The adenoid cystic/cribriform component showed pseudoluminal spaces containing eosinophilic secretions, while the basaloid component consisted of closely packed tumor cells arranged in solid nests and cords. (**B**–**F**) Immunohistochemical staining revealed diffuse positivity for CK5/6 (**B**) and p63 (**C**), supporting the basal cell phenotype. In contrast, tumor cells showed negative staining for PSA (**D**) and NKX3.1 (**E**), indicating the absence of conventional prostatic luminal differentiation. Ki-67 staining demonstrated an increased proliferative index (**F**). All images were obtained from the same representative tumor area (specimen ID: GX2511147-20HE-tj, left lobe, block #2) at the original magnification of ×200. Scale bars: 100 μm.

Immunohistochemical analysis of the radical prostatectomy specimen showed diffuse positivity for p63, CK5/6, and p40, with focal CK7 expression (Figure 4; Table 2). PSA, NKX3.1, and AR (androgen receptor) were negative, and the Ki-67 proliferation index was approximately 30%. The discordant PSA expression between the initial transurethral resection specimen and the radical prostatectomy specimen was considered to be related to the heterogeneous immunophenotypic characteristics of prostatic adenoid cystic/basal cell carcinoma and differences in tumor sampling between the two specimens.

Morphology and immunophenotypic characteristics were consistent with prostatic ACC/BCC.

### 2.6. FISH and Molecular Diagnosis

**Figure 5:**
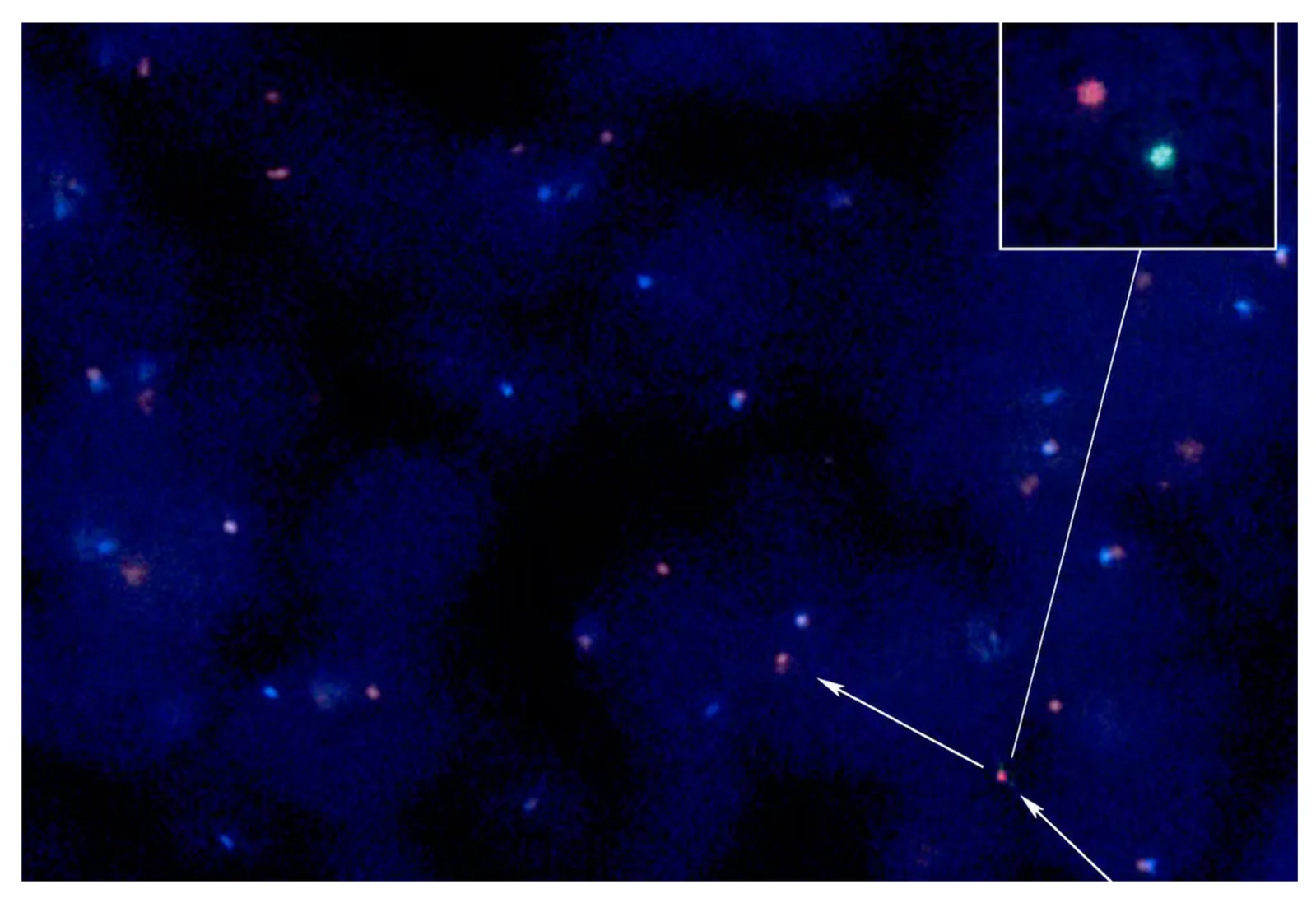

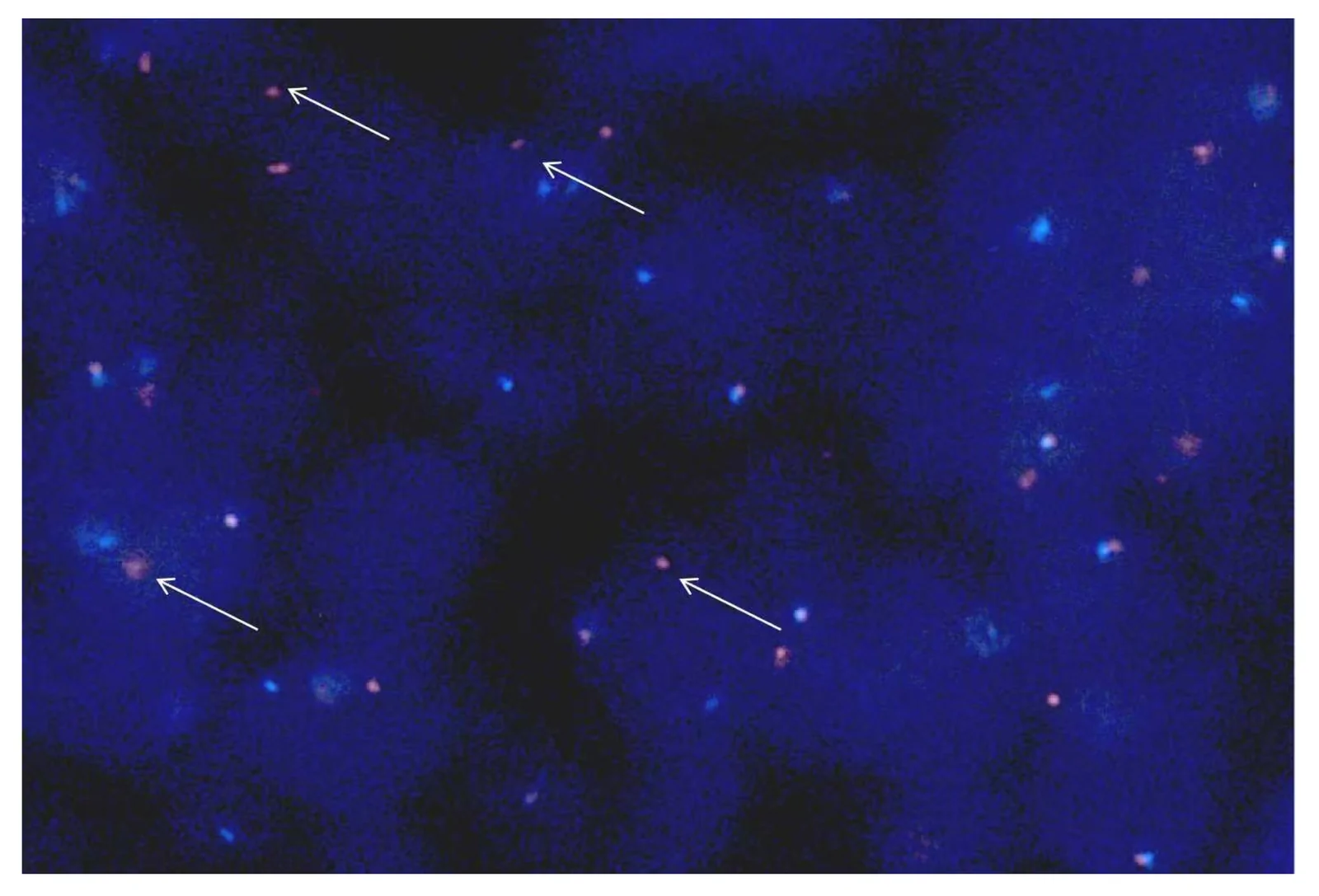
Fluorescence in situ hybridization analysis of MYB rearrangement. Representative tumor nuclei showing an abnormal 1R1G1F signal pattern (one red signal, one green signal, and one fused signal) are indicated by white arrows. The enlarged inset demonstrates the representative separated red and green signals consistent with MYB locus rearrangement.

To further clarify the molecular classification, MYB rearrangement was assessed using an MYB (6q23) dual-color break-apart probe (product code F.01247-01; GSP MYB [Centromere]/GSP MYB [Telomere]; Guangzhou LBP Medicine Science & Technology Co., Ltd., Guangzhou, China). A total of 200 tumor nuclei within the selected tumor area were evaluated. A typical 1R1G1F abnormal signal pattern, consisting of one each of red, green, and fused signals, was identified in 40% of the assessed tumor nuclei, exceeding the laboratory-defined positivity threshold of 15% [4,6,7]. The MYB rearrangement assay was performed on formalin-fixed paraffin-embedded tumor sections after routine deparaffinization, followed by protease pretreatment, probe hybridization, post-hybridization washing, and fluorescence signal detection according to the manufacturer’s recommended protocol. Fluorescence signals were evaluated using a fluorescence microscope equipped with appropriate filter sets for dual-color detection, and signal patterns were interpreted according to the manufacturer’s scoring criteria for break-apart FISH assays. The 15% positivity threshold was established according to the validated cutoff applied in our laboratory for MYB break-apart FISH interpretation, with consideration of background split signals observed in normal control tissues and routine assay performance evaluation. The tumor was therefore considered positive for MYB rearrangement (Figure 5).

Final diagnosis:

Prostatic adenoid cystic/basal cell carcinoma with MYB rearrangement, pT3aN0M0, with perineural invasion, focal bladder neck involvement, negative surgical margins, and absence of pelvic lymph node metastasis (0/15). The N0 category was based on the postoperative pathological examination, while M0 was assigned because the preoperative chest, abdominal, skeletal, and PET/CT examinations showed no distant metastasis.

### 2.7. Treatment Course and Short-Term Follow-Up

The patient had an uneventful postoperative recovery and was discharged. An indwelling urinary catheter was placed during surgery and was removed after urinary function had recovered satisfactorily, with only minimal leakage.

Notably, at the initial stage of treatment, the patient was presumed to have “high-risk prostatic adenocarcinoma” and was given androgen deprivation therapy combined with a next-generation AR inhibitor. Following integrated pathological and molecular reassessment, including detection of MYB rearrangement via FISH, the tumor was reclassified as prostatic ACC/BCC rather than conventional prostatic adenocarcinoma, prompting the multidisciplinary team to reconsider the initial management approach. Given the AR-negative immunophenotype and the revised diagnosis, continued androgen-directed therapy was considered unlikely to provide substantial clinical benefit. Therefore, goserelin and enzalutamide were discontinued on 27 September 2025, while the remaining related medications were subsequently withdrawn after a thorough discussion with the patient.

Previous clinical experience with ACC arose at other anatomical sites, including the head and neck and salivary glands [4], where platinum complexes and taxanes have achieved objective responses or stable disease control in some patients with recurrence or metastasis after initial treatment [8]. This was considered contextual information during multidisciplinary discussion, although its applicability to localized prostatic ACC/BCC remains uncertain.

Considering the high-risk pathological features in this case, including pT3a stage, perineural invasion, and an elevated Ki-67 index [1], as well as the overall condition of the patient and the absence of an established disease-specific adjuvant standard, a postoperative adjuvant regimen of paclitaxel plus carboplatin was selected as an individualized empirical treatment after multidisciplinary discussion. The patient was 162 cm tall and weighed 52 kg, corresponding to a calculated body surface area (BSA) of 1.50 m^2^, which was used for dose calculation. Paclitaxel (135 mg/m^2^) and carboplatin (AUC 5) were administered on day 1 of each 3-week cycle for a total of 6 cycles. Treatment was generally well tolerated, with grade 1 gastrointestinal symptoms, grade 1 myalgia, and grade 1–2 neutropenia observed during chemotherapy. Additionally, other therapeutic approaches, such as VEGFR inhibitors, may represent potential options for future investigation or clinical consideration regarding disease progression [9].

At the most recent follow-up in June 2026, the patient’s ECOG performance status remained stable. Follow-up chest and pelvic CT showed no definite evidence of local recurrence or distant metastasis. Serum TPSA and FPSA remained suppressed at <0.010 ng/mL and <0.01 ng/mL, respectively. However, considering the PSA-negative immunophenotype of the tumor and the preceding androgen deprivation therapy, PSA levels were considered insufficient to independently reflect tumor status and were therefore interpreted together with clinical evaluation and imaging findings. The patient completed six cycles of adjuvant chemotherapy as scheduled and tolerated treatment well.

## 3. Discussion

Adenoid cystic/basal cell carcinoma of the prostate gland belongs to the category of prostatic adenoid cystic carcinoma. The reported ages of onset range from 42 to 93 years. Patients generally present with lower urinary tract obstruction, and serum PSA levels are generally below the upper limit of the normal range; however, elevations may be seen in conjunction with acinar adenocarcinoma. In most cases, there are varying degrees of adeno-cystic/cribriform structures with condensed secretions and basaloid nests. The stroma frequently shows a desmoplastic reaction.

Histologically, there are five main differences between prostatic adenoid cystic (basal cell) carcinoma and florid basal cell hyperplasia, as follows:

1. Cytological atypia;
2. Infiltrative growth pattern within the stroma and among normal prostatic acini;
3. Perineural invasion;
4. Tumor necrosis (occurred in a few cases);
5. Extraprostatic extension.

Immunophenotypically, the tumor cells of prostatic ACC/BCC exhibit diffuse BCL2 expression and a high Ki-67 proliferative index compared with normal tissue; thus, they can be distinguished from florid basal cell hyperplasia.

Recent molecular studies have shown that, among some cases of prostatic basal cell carcinomas, there are specific gene fusions—including MSMB-NCOA4 (microseminoprotein beta-nuclear receptor coactivator 4) fusions [10]—which indicate that these tumors might have different driving molecular mechanisms from the conventional AR-axis-driven adenocarcinoma model.

Prostatic ACC/BCC also needs to be distinguished from urothelial cancer, high-grade acinar carcinoma, neuroendocrine carcinoma, and metastatic adenoid cystic carcinoma that has arisen elsewhere (such as in the bulbourethral gland) at the primary site.

Prostatic adenoid cystic (basal cell) carcinoma is an aggressive tumor, and definitive therapy must be administered. After radical prostatectomy, around 44–71% of patients experience metastasis to the extrapelvic area. A rate of distant metastasis between 14% and 29% has been reported, with approximately half of the patients eventually succumbing to the disease.

### 3.1. From Presumed Conventional Prostate Cancer to Prostatic ACC/BCC: The Importance of Diagnostic Reclassification

The misalignment between conventional prostate cancer and prostatic ACC/BCC principally arises in the three following aspects. Morphologically, tumor cells are mainly of basaloid cribriform/solid type, often accompanied by eosinophilic or mucinous luminal components, and conventional acinar Gleason grading is inapplicable.

Immunohistochemically, basal cell markers (p63, CK5/6, and p40) are uniformly positive, while PSA, NKX3.1, and AR are negative or weakly expressed. Regarding the Ki-67 index, a value of 30% or higher distinguishes these lesions from their benign counterparts and suggests malignancy; this finding aligns with earlier studies reporting elevated proliferation rates in ACC and BCC [3,11]. This immunophenotype is inconsistent with adenocarcinoma lineage.

At the molecular level, a portion of cases have MYB-NFIB fusions or rearrangements, which are considered molecular signatures of ACC. These genetic alterations may activate downstream targets, such as BCL2 and MYC [12], thereby further promoting tumor growth [13]. Note that MYB rearrangement is a characteristic change but is not universally present; other molecular abnormalities, such as MSMB-NCOA4 fusion [10], may be present to some extent.

In this case, the patient initially presented with urinary obstruction. The TUPKP specimen was misinterpreted as poorly differentiated carcinoma, and ADT (androgen deprivation therapy) was thus started prematurely. This sequence of events suggests a lack of awareness among clinicians regarding this rare tumor entity.

It must be noted that, for the diagnosis of prostatic ACC/BCC, more comprehensive diagnostic evidence is needed in addition to morphological changes, as outlined above; confirmatory laboratory or immunohistochemistry tests need to be conducted simultaneously; and a comprehensive examination should be performed through molecular analysis. The immunophenotypic characteristics of this case (negative for PSA and AR; positive for P63 and CK5/6) were in line with those observed in previous publications. Pathological consultation raised the possibility of prostatic ACC/BCC, and subsequent FISH analysis confirmed MYB rearrangement, supporting the revised diagnosis. This molecular reclassification clarified the tumor diagnosis and provided a basis for reassessing subsequent clinical management.

### 3.2. MYB Rearrangement

#### Diagnostic Support for Tumor Classification

Bishop et al. have identified MYB rearrangements in a subset of prostatic basal cell carcinomas and demonstrated molecular similarities with salivary gland ACC [14]; in addition, MYB functions as a transcription factor that regulates downstream gene expression through interactions with transcriptional coactivators such as CBP/p300. Magers et al. also verified that approximately 50 percent of prostatic BCCs have a MYB-NFIB fusion gene, and the proportion of positive cases in this group is higher. Notably, these tumors often have adenoid cystic-like morphology and perineural invasion.

In this context, the fifth edition of the WHO classification referred to this type as “prostatic adenoid cystic carcinoma/basal cell carcinoma” to distinguish it from cutaneous basal cell carcinoma.

FISH confirmation of MYB rearrangement in this case completed the diagnostic process from morphological suspicion to molecular confirmation, providing additional molecular support for tumor classification. Importantly, the molecular finding itself was not considered a therapeutic biomarker and did not establish an indication for systemic therapy. Rather, reclassification of the tumor as prostatic ACC/BCC prompted reassessment of the initial management approach, which was based on the treatment paradigm for conventional prostate adenocarcinoma. Given the absence of an established disease-specific treatment standard, subsequent management decisions were individualized through multidisciplinary discussion. Compared with previous reports, the most distinctive feature of this case is the explicit record of inappropriate ADT caused by a misdiagnosis at the beginning, which was then decisively discontinued after molecular diagnosis provided evidence of MYB rearrangement or AR negativity. Considering the high-risk pathological features of this case and the absence of an established adjuvant treatment standard, an adjuvant chemotherapy regimen consisting of paclitaxel plus carboplatin was administered as an individualized empirical decision after multidisciplinary discussion [15].

This case highlights that tumors with atypical immunophenotypic features, such as PSA/AR-negative prostatic ACC/BCC, require accurate diagnostic reassessment rather than classification solely according to conventional prostatic adenocarcinoma paradigms.

### 3.3. Reconstruction of Therapeutic Strategy and Literature Comparison

As prostatic ACC/BCC is exceptionally rare, evidence regarding its treatment and long-term prognosis is mainly derived from case reports and small retrospective series. Experience from ACC arising at other anatomical sites, particularly the salivary glands and head and neck, may therefore provide useful clinical context. The available epidemiological data, treatment approaches, and reported outcomes are summarized in Supplementary Table S1. Nevertheless, differences in anatomical site and disease setting should be considered when interpreting these data.

#### 3.3.1. Localized ACC/BCC

##### Local Control as the Foundation and Consideration of Systemic Therapy in High-Risk Disease

Most previous cases involved radical surgery or radiotherapy as treatment methods at this stage; however, local recurrence and distant metastasis are common and tend to occur more frequently in cases involving larger tumor size, positive surgical margins, or a high Ki-67 index.

For previous cases of salivary gland and other head and neck ACC, platinum-based regimens have achieved objective response rates of 10–30% with relatively high disease control rates in recurrence/metastasis situations. Taxanes and platinum-containing drugs have also shown synergistic antitumor activity in some preclinical studies.

Nevertheless, evidence for early systemic therapy in localized prostatic ACC/BCC remains very limited. Experience with platinum-based regimens in recurrent or metastatic ACC may inform treatment considerations for selected high-risk patients but does not establish a role for adjuvant chemotherapy in this setting. In the present case, paclitaxel plus carboplatin after radical resection was used on an individualized, empirical basis in the absence of an established disease-specific adjuvant standard.

Furthermore, due to the presence of bladder neck and perineural invasions during surgery, radiation therapy after surgery can increase the extent of control at this site. However, this has not yet been proven conclusively by clinical trial data [16,17]. In this context, the present case illustrates the importance of considering individualized systemic therapy in addition to local control for high-risk prostatic adenoid cystic carcinoma/basal cell carcinoma and may provide insights for future clinical investigations.

#### 3.3.2. Long-Term Planning

##### VEGFR Inhibitors and Clinical Trials

Systematic reviews indicate that the disease control rate and progression-free survival are relatively high for patients undergoing VEGFR inhibitor treatment (such as Lenvatinib and axitinib), suggesting a potential therapeutic option for subsequent treatment to extend life [9,18,19].

If metastasis occurs or the disease progresses after chemotherapy, VEGFR inhibitors or relevant basket trials may be considered [20].

## 4. Conclusions

In summary, this case systematically highlights the diagnostic difficulties and individualized management considerations for prostatic adenoid cystic/basal cell carcinoma (ACC/BCC), as a rare tumor type.

For PSA-negative/AR-negative prostatic tumors with atypical morphology, increased vigilance must be exercised. Integrating histochemical, immunohistochemical, and molecular biology techniques, including fluorescence in situ hybridization testing (FISH), is essential to avoid the inappropriate use of ADT, thereby preventing misdiagnosis and therapeutic errors.

A notable feature of this case is the adjustment of clinical management after the tumor was reclassified as MYB-rearranged prostatic ACC/BCC. The AR-negative phenotype, low/negative PSA expression, and high-risk pathological features informed discontinuation of conventional endocrine therapy and the subsequent individualized management decision, including radical surgery followed by adjuvant paclitaxel plus carboplatin. This individualized treatment course may provide a useful clinical reference for similar rare cases, although its long-term value requires further follow-up and additional evidence.

As a single case report, there are limitations to this study. Although paclitaxel plus carboplatin has been reported in ACC, the available systemic treatment evidence is derived predominantly from recurrent or metastatic disease, and evidence supporting its use as postoperative adjuvant therapy for completely resected prostatic ACC/BCC remains insufficient. While clinical experience from ACC arising at other anatomical sites provided background information for considering this regimen, its application in the current postoperative setting constitutes a clinical extrapolation that necessitates further validation. In this context, the present case provides a clinically relevant observation that may broaden the therapeutic perspective for this rare malignancy, but should not be interpreted as establishing the efficacy of paclitaxel plus carboplatin or defining a validated adjuvant treatment strategy. The relatively short follow-up period is another major limitation. At the most recent assessment, approximately 9.5 months post-surgery, no definite recurrence or metastasis was observed. However, this short-term observation cannot establish treatment efficacy or validate a successful management model, and longer follow-up and further evidence are required.

Future multi-institutional collaborations must accumulate a larger number of cases to determine the most suitable adjuvant or systemic treatment strategies for this rare tumor. Future research should explore potential therapeutic targets (such as VEGFR) to identify novel therapeutic options for patients with advanced or recurrent disease.

## Figures and Tables

**Table 1:** Baseline laboratory findings at admission.

Category	Parameter	Result	Reference Range
Prostate-specific antigen	Total PSA (TPSA), ng/mL	1.077	0–4.0
	Free PSA (FPSA), ng/mL	0.05	0–0.93
Hematology	White blood cell count, ×10^9^/L	5.85	3.5–9.5
	Neutrophils, ×10^9^/L	3.40	1.8–6.3
	Lymphocytes, ×10^9^/L	1.94	1.1–3.2
	Hemoglobin, g/L	130	130–175
	Hematocrit, %	38.4	40–50
	Platelet count, ×10^9^/L	326	125–350
Coagulation	Prothrombin time, s	11.4	9–13
	International normalized ratio	0.97	0.85–1.15
	Activated partial thromboplastin time, s	30.5	23.3–32.5
	Fibrinogen, g/L	3.33	2.00–4.00
	D-dimer, μg/mL	0.19	0–0.5
Liver function	Total protein, g/L	67.7	65–85
	Albumin, g/L	43.1	40–55
	Total bilirubin, μmol/L	19.0	0–23
	Alanine aminotransferase, U/L	4.4	9–50
	Aspartate aminotransferase, U/L	11.5	15–40
	Alkaline phosphatase, U/L	76	45–125
	γ-Glutamyl transferase, U/L	20.1	10–60
Renal function	Blood urea nitrogen, mmol/L	5.47	3.60–9.50
	Serum creatinine, μmol/L	57.7	57.0–111.0
	Cystatin C, mg/L	1.10	0.50–1.07
	Estimated glomerular filtration rate, mL/min/1.73 m^2^	106	>90
Electrolytes and metabolism	Potassium, mmol/L	3.84	3.50–5.30
	Natrium, mmol/L	140.0	137.0–147.0
	Calcium, mmol/L	2.27	2.11–2.52
	Fasting glucose, mmol/L	6.37	3.89–6.11

1. Note: All measurements were obtained on 15 August 2025, before radical prostatectomy. Minor isolated deviations in hematocrit, aminotransferase levels, cystatin C, and fasting glucose were not considered clinically significant and did not preclude surgery. 2. Abbreviations: PSA, prostate-specific antigen; TPSA, total prostate-specific antigen; FPSA, free prostate-specific antigen.

**Table 2:** Immunohistochemical and molecular findings.

(A) Immunohistochemistry			
Marker	Result		Interpretation
p63	Positive (nuclear)		Basal/myoepithelial differentiation
CK5/6	Positive (cytoplasmic)		Basal-type cytokeratin
p40	Positive (nuclear)		Basal/squamoid marker
34βE12 (HMWCK)	Positive (cytoplasmic)		Basal cell phenotype
CK7	Focal positive (cytoplasmic)		Supports luminal/ductal differentiation in a subset of tumor cells
PSA	Negative		Not supportive of conventional acinar adenocarcinoma
NKX3.1	Negative (nuclear)		Further argues against a typical prostatic acinar phenotype
AR	Negative (nuclear)		Consistent with an AR-independent phenotype
ERG	Negative (nuclear)		No evidence of ERG overexpression (TMPRSS2–ERG–type)
p504s (AMACR)	Negative		AMACR negative, not typical for conventional acinar adenocarcinoma
PTEN	Heterogeneous (+/−, per report)		Suggests intratumoral heterogeneity; interpret with caution
Ki-67	~30%		Proliferation index
**(B) Molecular testing.**			
**Assay**	**Target**	**Result**	**Key details**
Break-apart FISH	MYB rearrangement	Positive	Tumor area; 200 nuclei analyzed; split-signal pattern (1R1G1F) in 40% of cells, exceeding the laboratory-defined cutoff, consistent with MYB rearrangement and supporting ACC lineage.

Abbreviations: HMWCK, high-molecular-weight cytokeratin; AMACR, alpha-methylacyl-CoA racemase; FISH, fluorescence in situ hybridization; VAF, variant allele frequency.

## Data Availability

The data supporting the findings of this case report are included within the article. Additional raw clinical data are not publicly available to protect patient privacy and confidentiality.

## Supplementary Materials

The following supporting information can be downloaded at: https://www.mdpi.com/article/10.3390/curroncol33090527/s1, Table S1: Epidemiology, treatment, and reported outcomes of prostatic ACC/BCC and ACC at selected anatomical sites.

## References

[1] Mohanty S.K., Lobo A., Cheng L. (2022). The 2022 revision of the World Health Organization classification of tumors of the urinary system and male genital organs: Advances and challenges. Hum. Pathol..

[2] Netto G.J., Amin M.B., Berney D.M., Compérat E.M., Gill A.J., Hartmann A., Menon S., Raspollini M.R., Rubin M.A., Srigley J.R. (2022). The 2022 World Health Organization Classification of Tumors of the Urinary System and Male Genital Organs—Part B: Prostate and Urinary Tract Tumors. Eur. Urol..

[3] Taskovska M., Frelih M., Smrkolj T., Volavšek M. (2023). Basal Cell Carcinoma of the Prostate Misdiagnosed as High-Grade Urothelial Cancer–A Case Report of a Diagnostic Pitfall. Res. Rep. Urol..

[4] Persson M., Andersson M.K., Mitani Y., Brandwein-Weber M.S., Frierson H.F., Moskaluk C., Fonseca I., Ferrarotto R., Boecker W., Loening T. (2022). Rearrangements, Expression, and Clinical Significance of MYB and MYBL1 in Adenoid Cystic Carcinoma: A Multi-Institutional Study. Cancers.

[5] Magers M.J., Iczkowski K.A., Montironi R., Grignon D.J., Zhang S., Williamson S.R., Yang X., Wang M., Osunkoya A.O., Lopez-Beltran A. (2019). MYB-NFIB gene fusion in prostatic basal cell carcinoma: Clinicopathologic correlates and comparison with basal cell adenoma and florid basal cell hyperplasia. Mod. Pathol..

[6] Rettig E.M., Tan M., Ling S., Yonescu R., Bishop J.A., Fakhry C., Ha P.K. (2015). MYB rearrangement and clinicopathologic characteristics in head and neck adenoid cystic carcinoma. Laryngoscope.

[7] Han J., Zhang C., Gu T., Yang X., Hu L., Tian Z., Li J., Zhang C. (2019). Analysis of clinicopathological characteristics, MYB rearrangement and prognostic factors in salivary adenoid cystic carcinoma. Oncol. Lett..

[8] Prost D., Iseas S., Gatineau M., Adam J., Cavalieri S., Bergamini C., Licitra L., Raymond É. (2024). Systemic treatments in recurrent or metastatic salivary gland cancer: A systematic review. ESMO Open.

[9] Hoff C.O., Manzi J., Lazar Neto F., Ferrarotto R. (2024). Vascular Endothelial Growth Factor Receptor Inhibitors for Recurrent or Metastatic Adenoid Cystic Carcinoma: A Systematic Review and Meta-Analysis. JAMA Otolaryngol. Head Neck Surg..

[10] Pedersen V., Petersen K.S., Brasso K., Østrup O., Loya A.C. (2021). Basal Cell Carcinoma of Prostate with MSMB–NCOA4 Fusion and a Probable Basal Cell Carcinoma In Situ: Case Report. Int. J. Surg. Pathol..

[11] Low J.-Y., Ko M., Hanratty B., Patel R.A., Bhamidipati A., Heaphy C.M., Sayar E., Lee J.K., Li S., De Marzo A.M. (2022). Genomic Characterization of Prostatic Basal Cell Carcinoma. Am. J. Pathol..

[12] Wei S., Pei J., Zhang P.J.L. (2025). Molecular pathology of adenoid cystic carcinoma. Histol. Histopathol..

[13] Mishra V., Singh A., Korzinkin M., Cheng X., Wing C., Sarkisova V., Koppayi A.L., Pogorelskaya A., Glushchenko O., Sundaresan M. (2025). PRMT5 inhibition has a potent anti-tumor activity against adenoid cystic carcinoma of salivary glands. J. Exp. Clin. Cancer Res..

[14] Bishop J.A., Yonescu R., Epstein J.I., Westra W.H. (2015). A subset of prostatic basal cell carcinomas harbor the MYB rearrangement of adenoid cystic carcinoma. Hum. Pathol..

[15] Zhou J., Zhao G., Wang S., Li N. (2024). Systemic therapy in the management of metastatic or locally recurrent adenoid cystic carcinoma of the salivary glands: A systematic review of the last decade. Br. J. Cancer.

[16] Cozzi S., Bardoscia L., Najafi M., Botti A., Blandino G., Augugliaro M., Manicone M., Iori F., Giaccherini L., Sardaro A. (2022). Adenoid Cystic Carcinoma/Basal Cell Carcinoma of the Prostate: Overview and Update on Rare Prostate Cancer Subtypes. Curr. Oncol..

[17] Dong S., Liu Q., Xu Z., Wang H. (2020). An Unusual Case of Metastatic Basal Cell Carcinoma of the Prostate: A Case Report and Literature Review. Front. Oncol..

[18] Tchekmedyian V., Sherman E.J., Dunn L., Tran C., Baxi S., Katabi N., Antonescu C.R., Ostrovnaya I., Haque S.S., Pfister D.G. (2019). Phase II Study of Lenvatinib in Patients with Progressive, Recurrent or Metastatic Adenoid Cystic Carcinoma. J. Clin. Oncol..

[19] Kang E.J., Ahn M.-J., Ock C.-Y., Lee K.-W., Kwon J.H., Yang Y., Choi Y.H., Kim M.K., Ji J.H., Yun T. (2021). Randomized Phase II Study of Axitinib versus Observation in Patients with Recurred or Metastatic Adenoid Cystic Carcinoma. Clin. Cancer Res..

[20] Ferrarotto R., Sousa L.G., Feng L., Mott F., Blumenschein G., Altan M., Bell D., Bonini F., Li K., Marques-Piubelli M.L. (2023). Phase II Clinical Trial of Axitinib and Avelumab in Patients with Recurrent/Metastatic Adenoid Cystic Carcinoma. J. Clin. Oncol..

