# Supplementary material for: Case of MYB-Rearranged Prostatic Adenoid Cystic Carcinoma
Source: Curr Oncol. 2026 Sep 1;33(9):527. doi: 10.3390/curroncol33090527 (PMC13605576; doi:10.3390/curroncol33090527)
Supplement: Supplementary file 1 [file curroncol-33-00527-s001.zip › curroncol-4379454-supplementary.pdf]

Supplementary Table S1. Epidemiology, treatment, and reported outcomes of prostatic ACC/BCC and ACC at selected anatomical sites

Tumour setting	Evidence base	Main treatment	Key quantitative outcomes
Prostatic ACC/BCC[a]	106 patients; 33 reports/series	Resectable disease: radical surgery when feasible; no established adjuvant or systemic standard	Treatment among 106 reported patients: transurethral resection, 36.5%; radical prostatectomy, 16.4%; radical prostatectomy plus postoperative radiotherapy, 10.36%; systemic therapy, 5.7%. Outcomes among 90 patients with available follow-up: NED, 45.5%; alive with disease, 23.3%; progression/metastasis, 26.6%; disease-specific death, 4.4%.
Salivary gland/head-and-neck ACC[bc]	17,497 patients; 57 studies; ASCO guideline; phase II trials	Resectable disease: surgery followed by postoperative radiotherapy. Progressive recurrent/metastatic disease: individualized systemic therapy, including VEGFR-directed agents	OS: 5 years, 73.8%; 10 years, 48.5%; 15 years, 42.3%; 20 years, 26.5%. Mean time to distant metastasis, 34.8 months; local recurrence, 31.3 months. Lenvatinib: ORR, 15.6%; median PFS, 17.5 months. Axitinib plus avelumab: ORR, 18%; median PFS, 7.3 months.
Primary tracheal ACC[d]	1,488 patients; 19 studies	Surgery was the first-choice treatment in 74.5%; adjuvant radiotherapy was used in 56.7%	Pooled 5-year OS, 65%; reported study range, 32%–96%.

Abbreviations: ACC, adenoid cystic carcinoma; BCC, basal cell carcinoma; NED, no evidence of disease; ORR, objective response rate; OS, overall survival; PFS, progression-free survival; VEGFR, vascular endothelial growth factor receptor.

a. Cozzi S, Bardoscia L, Najafi M, Botti A, Blandino G, Augugliaro M, et al. Adenoid cystic carcinoma/basal cell carcinoma of the prostate: overview and update on rare prostate cancer subtypes. Curr Oncol. 2022;29(3):1866–1876. doi:10.3390/curroncol29030152.

b. Lavareze L, de Carvalho Kimura T, Cacita N, et al. Survival outcomes in adenoid cystic carcinoma of the head and neck: a systematic review and meta-analysis. Head Neck. 2025;47(5):1541–1553. doi:10.1002/hed.28132. Geiger JL, Ismaila N, Beadle B, et al. Management of salivary gland malignancy: ASCO guideline. J Clin Oncol. 2021;39(17):1909–1941. doi:10.1200/JCO.21.00449.

c. Tchekmedyian V, Sherman EJ, Dunn L, et al. Phase II study of lenvatinib in patients with progressive, recurrent or metastatic adenoid cystic carcinoma. J Clin Oncol. 2019;37(18):1529–1537. doi:10.1200/JCO.18.01859. Ferrarotto R, Sousa LG, Feng L, et al. Phase II clinical trial of axitinib and avelumab in patients with recurrent/metastatic adenoid cystic carcinoma. J Clin Oncol. 2023;41(15):2843–2851. doi:10.1200/JCO.22.02221.

d. Shi Y, Xue S, Zou J. Treatment and outcomes of primary tracheal adenoid cystic carcinoma: a systematic review. Cancer Manag Res. 2025;17:697–710.

Note: Because treatment evidence for prostatic ACC/BCC remains limited to case reports and small series, data from other anatomical sites are presented for clinical context rather than as directly transferable treatment standards.
